# Effect of physisorption and chemisorption of water on resonant modes of rolled-up tubular microcavities

**DOI:** 10.1186/1556-276X-8-531

**Published:** 2013-12-18

**Authors:** Jian Zhong, Jiao Wang, Gaoshan Huang, Guoliang Yuan, Yongfeng Mei

**Affiliations:** 1 Department of Materials Science and Engineering, Nanjing University of Science and Technology, Nanjing 210094, People’s Republic of China; 2 Department of Materials Science, Fudan University, Shanghai 200433, People’s Republic of China

**Keywords:** Microcavity, Coating, Mode shift, Desorption, Detection

## Abstract

Both blue- and redshifts of resonant modes are observed in the rolled-up Y_2_O_3_/ZrO_2_ tubular microcavity during a conformal oxide coating process. Our investigation based on spectral analyses suggests that there are two competitive processes during coating: desorption of both chemically and physically absorbed water molecules and increase of the tube wall thickness. The redshift is due to the increase of the wall thickness and corresponding light confinement enhancement. On the other hand, desorption of water molecules by heating leads to a blueshift. The balance of these two factors produces the observed bi-directional shift of the modes while they both contribute to promoted quality factor after coating.

## Background

Optical microcavities with tubular geometry exhibit several advantages compared to other types of optical microcavities [1-4]. They naturally assume a hollow structure and are fully integrative into lab-on-chip systems [5]. In the past years, rolled-up tubular microcavities have been used as cell culture devices [6,7], microlasers [8,9], sensors [10], and so on. Especially, rolled-up microcavities with (ultra)thin wall thickness are sensitive to tiny alterations and modifications in the vicinity of the inner and outer tube wall surfaces [5]. Thus, the microcavities exhibit excellent potential applications as sensors in the fields of optoelectronics [11], biosensing [6,12], and integrated optofluidics [10,13]. Very recently, preliminary results concerning detection of dynamic molecular processes were demonstrated on a self-rolled-up SiO/SiO_2_ optical microcavity with sub-wavelength wall thickness [14]. In fact, the molecule absorption/desorption are quite complex processes, and their interaction with the evanescent field is even intricate, especially in the nanoscale. Before this sensing technique can be put into practical applications like other label-free methods, more work must be done to disclose the mechanism and to exhibit the general and diverse capability of the approach.

In this letter, we focus on the detection of physically and/or chemically absorbed water molecules by using a rolled-up tubular microcavity as a core component. The microcavities used in this work were prepared by releasing prestressed 33.5-nm-thick Y_2_O_3_/ZrO_2_ circular nanomembranes on photoresist sacrificial layers. The influence of surface composition (e.g., coating formed by atomic layer deposition (ALD) and water molecules absorbed from atmosphere) on the mode (including the sub-modes) positions as well as the *Q*-factors will be discussed on the basis of detailed spectral analyses. Different from a previous work [15], we conduct a much more meticulous ALD coating process and observe an unusual blueshift of the resonant mode in the present case. We find that the observation originates from the effects of both chemisorption and physisorption water molecules, suggesting a rather complicated nature of the interaction between the evanescent field and the surrounding environment.

## Methods

The bare Y_2_O_3_/ZrO_2_ tubular optical microcavities are prepared via self-rolled nanotechnology as described elsewhere [16]. These Y_2_O_3_/ZrO_2_ microtubes are uniformly coated with up to 150 monolayers (MLs) of HfO_2_ by ALD to tune the optical resonant modes [10]. Tetrakis(dimethylamino)hafnium (Hf[N(CH_3_)_2_]_4_) and H_2_O are used as precursor sources; pulse times for hafnium source and water source are both 15 ms per circle. The abovementioned two precursors react completely in each circle at 150°C and 30 Pa (N_2_ as the carrying gas) to obtain HfO_2_ coating layer on the wall of the microtube. The thickness of the HfO_2_ layer is approximately 2 Å/ML, which is calibrated using an atomic force microscope (AFM). After coating of every 10 HfO_2_ MLs, the sample is taken out and the microphotoluminescence (micro-PL) spectra (excitation wavelength 514 nm) are collected from the center spot of the microtube. All the optical measurements were carried out in the air at room temperature. Light emission from defect-related luminescent centers can circulate and interfere constructively in the circular cross section of the tubular microcavity forming stable resonance at certain wavelengths, noticed as an optical resonance mode [16,17].

## Results and discussion

**Figure 1:**
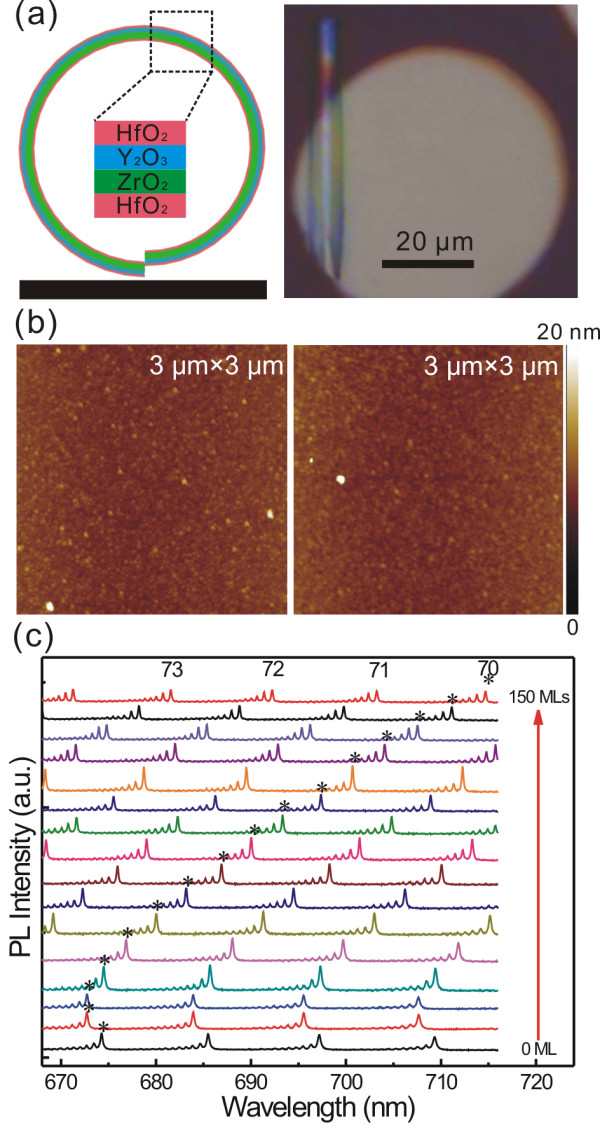
**Fabrication of the microtube and its typical PL spectra. (a)** Schematic diagram of the cross-sectional view of the microtube after HfO_2_ coating (left panel). The inset indicates the multilayer structure of the tube wall. The right panel shows the optical microscope image of a microtube with coating of 150 HfO_2_ MLs. **(b)** AFM images of the flat Y_2_O_3_/ZrO_2_ nanomembranes with (left panel) and without (right panel) coating of 150 HfO_2_ MLs. **(c)** Typical PL spectra collected from the center spot of the microtube with different HfO_2_ coatings (0 to 150 MLs with a step of 10 MLs). The marked (asterisk) modes' azimuthal numbers are *m* = 70.

The left part of Figure 1a schematically shows a cross-sectional view of the microtube, and both the inner and the outer surfaces of the tube walls are coated with the oxide layers. An optical microscope image of the microtube with a diameter of approximately 9 μm coated by 150 MLs of HfO_2_ is displayed in the right part of Figure 1a. The microtube is still transparent after this coating treatment, and the perfect tubular structure and directionality are obvious [6]. In addition, our AFM results indicate that the surfaces are quite smooth without significant variation in roughness after the ALD coating (Figure 1b). This feature suggests that the ALD coating process is quite suitable for tailoring the optical resonator and for microfluidic applications since the surface roughness will contribute remarkable light loss [17] and resistance in fluidics. Although there is no noticeable change in the morphology, the PL measurements show an interesting bi-directional change in the positions of optical modes. Figure 1c displays a series of PL spectra with coating from 0 to 150 MLs with a step of 10 MLs. Each PL spectrum exhibits several groups of sharp peaks which are known as optical modes [10,16], and the sub-modes in the group are previously ascribed to the axial confinement in the tubular microcavity [11,16]. With the increase of the number of the coating layers (i.e., the thickness of the HfO_2_ coating), all the modes shift to a shorter wavelength at the very beginning but then continuously move to a longer wavelength (Figure 1c).

**Figure 2:**
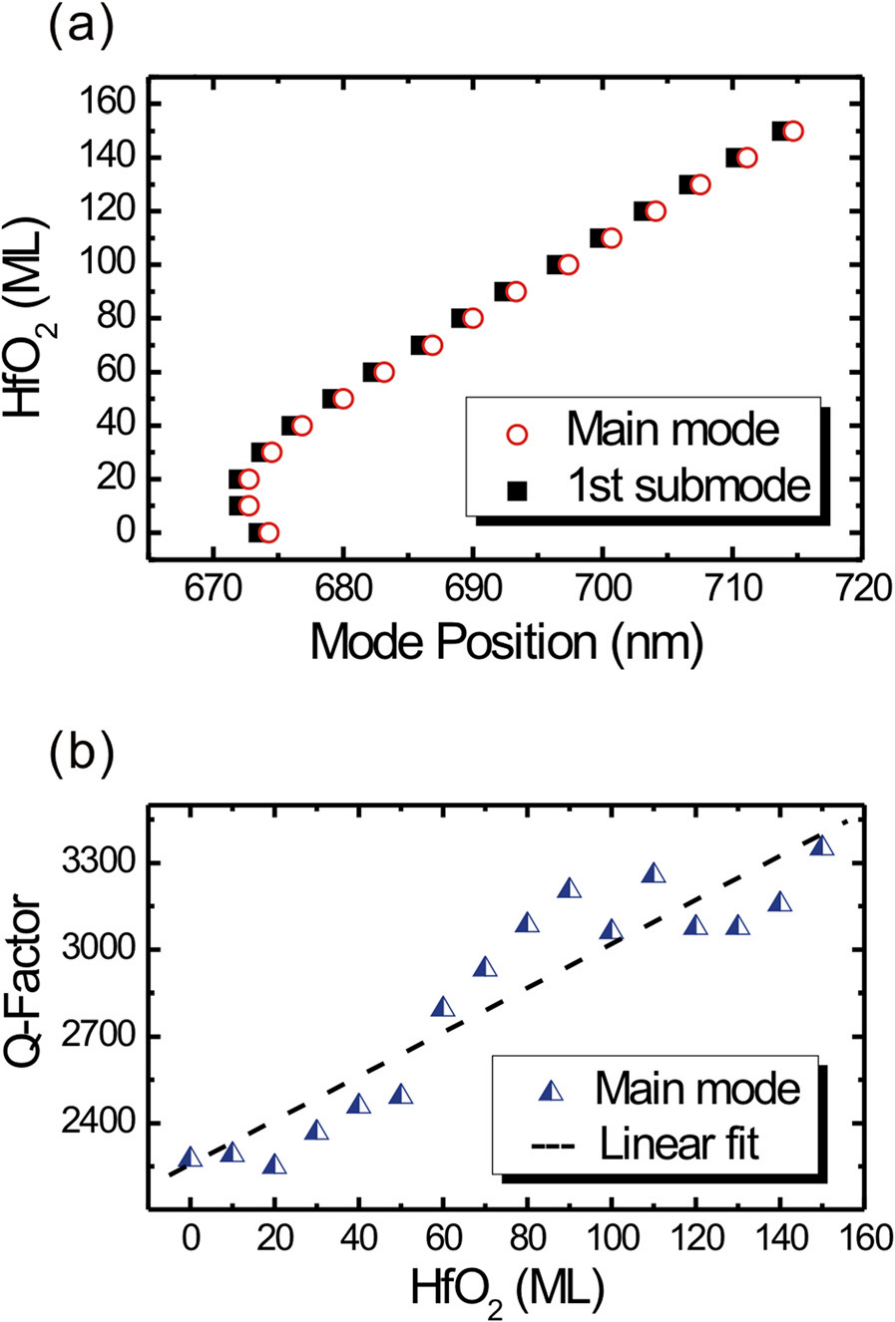
**Evolution of mode positions and *Q*-factors with increasing coating layers. (a)** Shift of mode (*m* = 70, main mode and first sub-mode) with increasing HfO_2_ coating layers. The dark squares and open circles represent the positions of the main mode and the first sub-mode, respectively. **(b)** Evolution of the *Q*-factor of mode (*m* = 70) with the coating layer. The triangles are the experimental results and the dashed line is the corresponding linear fit.

To make the results more intuitionistic, we extracted the positions of the mode with *m* = 70 (derived theoretically) and the corresponding first sub-mode and plotted the positions as a function of the number of coating layers, as shown in Figure 2a. One can see that both modes demonstrate the same shift tendency, indicating that this is not a coincidence. The key factor leading to this bi-directional shift influences not only the circular but also the axial propagations. The phenomenon has not been previously reported in a similar experiment with Al_2_O_3_ coating [15], and we will discuss the mechanism in the following paragraphs.

According to the literature, the mode positions show a strong relationship with the evanescent field and the surrounding medium [5,10], and the interaction of evanescent field with the absorption molecules on the wall of tubular microcavity leads to a detectable shift in the resonant frequency (i.e., mode position) [10,18] The previous experimental [15] and theoretical [19] results indicated that the resonant wavelength monotonically redshifts with increasing thickness of the high-refractive-index oxide (Al_2_O_3_ or HfO_2_) coating. In the present case, the modes show an obvious redshift with the HfO_2_ coating increasing from 20 to 150 MLs (Figures 1c and 2a), which fits well with the previous experimental results and theoretical prediction. However, when the HfO_2_ coating is less than 20 MLs, the mode positions show an unusual blueshift, which in any case suggests a change in the light's circulation and interference in the microcavity. To check the light confinement therein, we calculated the *Q*-factor using the formula *Q* = *λ*/∆*λ*, where *λ* and ∆*λ* denote the mode position and the full width at half maximum (FWHM) of the mode, respectively [16], and the results are plotted in Figure 2b. It is not surprising that as a consequence of the improved light confinement, the *Q*-factor appears to have a pronounced enhancement with increasing coating layers. However, the blueshift of modes in the case of a few coating layers ought to be related to other effects different from the increasing wall thickness.

**Figure 3:**
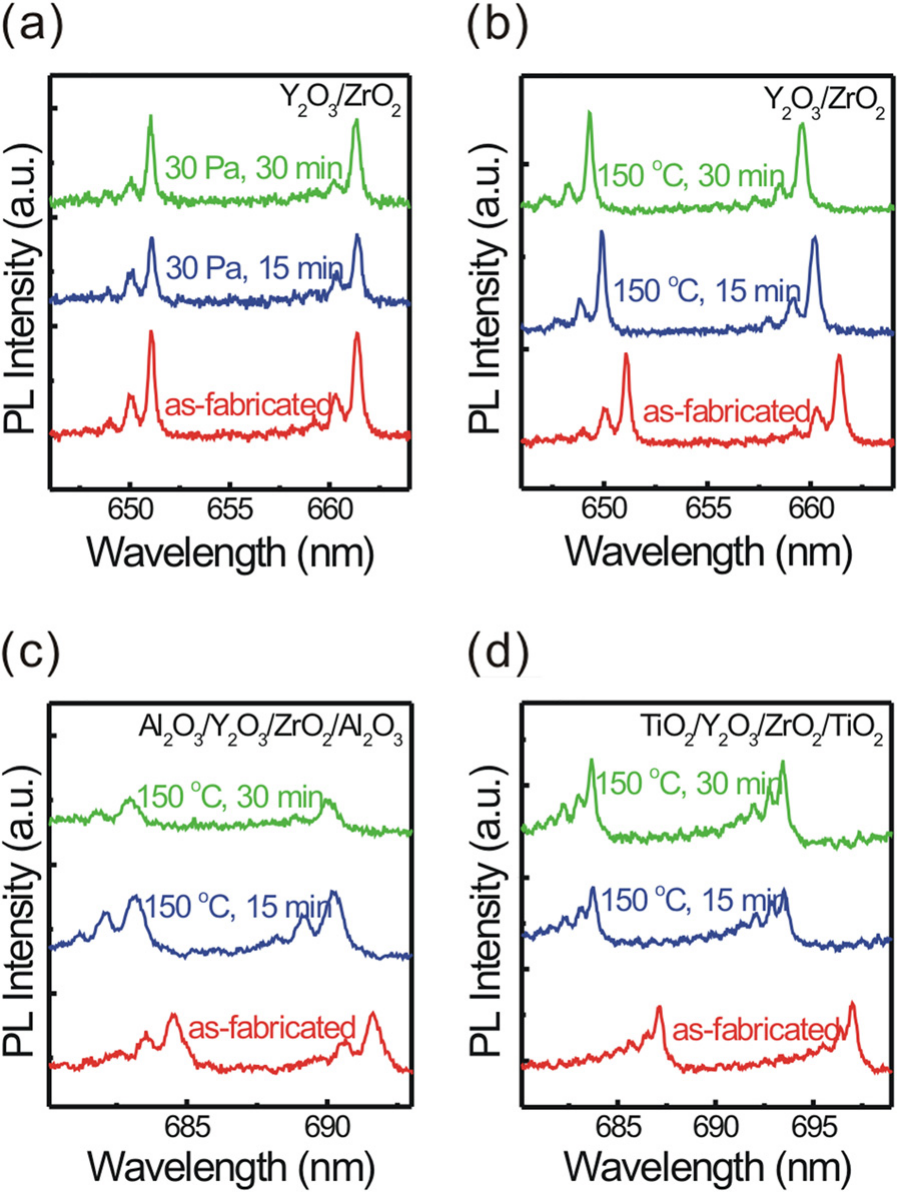
**PL spectra of microtubes with different coating layers after different treatments. (a, b)** PL spectra from the tubular microcavity after different treatments: **(a)** stored at 30 Pa and **(b)** heated at 150°C. **(c, d)** PL spectra from different tubular microcavities (reference samples) after heat treatment at 150°C: **(c)** microtube coated with 30-nm Al_2_O_3_ and **(d)** microtube coated with 30-nm TiO_2_.

We guess that the ALD process should be responsible for this unusual blueshift. Note that the process was carried out at 150°C and under vacuum. To go into more details, we checked the PL spectra of bared microtubes with different posttreatments (vacuum and heat treatment). Figure 3a,b shows the influence of vacuum and heat treatments on the mode positions, respectively. Compared with the vacuum, the heat treatment obviously plays an important role on the blueshift of the modes. For comparison purposes, microtubes coated with other oxide layers like Al_2_O_3_ and TiO_2_ were brought in, and we also measured their spectra after they were heated in air (see Figure 3c,d); all measurements were carried out in the air at room temperature. One can see that the modes always show a blueshift after the microtube was heated to 150°C, no matter the microtube is bare or coated with Al_2_O_3_/TiO_2_. In other words, the heating causes the modes to blueshift. In addition, we should stress that the ALD coating can make the microtube robust enough to stand repeated liquid washing [6], and thus, we can rule out the possibility of the blueshift to be connected with the structural deformation since the strengthened microtube should not deform while being heated. Thus, in such circumstance, the change in surface composition, especially the desorption of atmospheric water molecules, becomes a considerable influence element responsible for the blueshift because the surface modification leads to a change in the evanescent field and in turn alters the resonance [10,14,15,18,20]. Briefly, we can deduce that there are two competitive processes existing during ALD coating: the desorption of the water molecules makes the modes move towards a shorter wavelength [15] and the increase in the wall thickness causes a redshift of the modes. At the beginning of the coating, desorption of water is predominant because a remarkable blueshift can be observed but only a few oxide layers were deposited leading to a neglectable increase of wall thickness. When more HfO_2_ is coated on the tube surface, the coating layers play a more critical role and no more water molecules could be detached, eventually producing the redshift.

**Figure 4:**
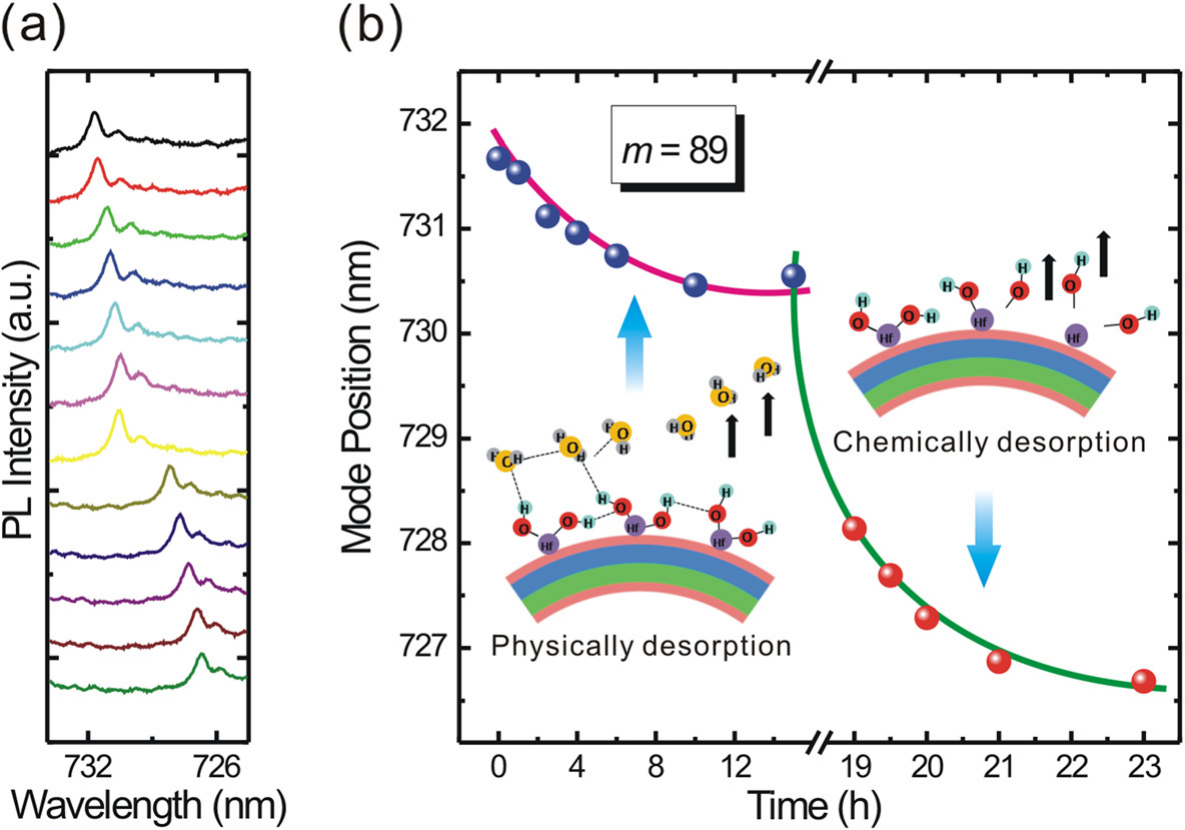
**Desorption process of absorbed water by drying and heating. (a)** A series of PL spectra of microtube after drying (top seven spectra, N_2_ flow at 50°C, 0 to 15 h) and heating (bottom five spectra, at 200°C under 30 Pa, N_2_ atmosphere, 15 to 23 h) as time goes on (from top to bottom). The measuring time points can be found in **(b)**. **(b)** The mode (*m* = 89) shifts as a function of treating time. The bottom-left and top-right insets schematically illustrate the desorption processes of physically and chemically absorbed water molecules, respectively.

As we know, the water will be adsorbed onto the tube wall both chemically and physically [18,20]. To investigate the influences from these two kinds of water molecules and the underneath mechanism, more experimental works have been carried out. An as-fabricated microtube (coated with 30-nm HfO_2_) was first dried in N_2_ flow at 50°C; PL spectra were measured in the air at room temperature immediately after every 30-min drying process (typical seven spectra are shown in the upper part of Figure 4a and the corresponding time points can be read from Figure 4b). The mode blueshifts approximately 1.2 nm in total and becomes steady after drying for 15 h, which is considered to be due to the removal of the physically absorbed water layer on the tube wall (see the diagram in the bottom-left inset in Figure 4b). The mode position finally becomes constant since the physically absorbed water molecules have been completely removed. Then, a heat treatment at 200°C under 30 Pa (N_2_ atmosphere) was introduced. PL spectra were also measured in the air at room temperature immediately after every 30-min heating treatment (typical five spectra are shown in the lower part of Figure 4a and the corresponding time points can be read from Figure 4b). The previous equilibrium is obviously broken, and a further blueshift from desorption of chemically absorbed water molecules can be detected. As reported in the literature [18], the microstructure of these chemically absorbed molecules is actually a layer of OH groups bound to the surface (see top-right inset in Figure 4b), which cannot be easily removed by a low-temperature drying process. After the microcavity was heated for more than 8 h, the mode position was maintained at a constant value again with a blueshift of approximately 3.8 nm (compared to dried microcavity). Figure 4b summarizes the mode position (*m* = 89) as a function of drying (N_2_ flow at 50°C, 0 to 15 h) and heating (200°C under 30 Pa, 15 to 23 h) time. The drying/heating treatments also indicate that desorption is not a rapid process but takes some time, which identifies with the results of the initial 20 MLs in Figure 2a. In short, this result demonstrates that the rolled-up microcavities with high sensitivity can be used as sensors for humidity detection.

Finally, we would like to discuss more about the influence of surface condition on the *Q*-factor. It is already well known that an oxide coating layer with high refractive index promotes an effective refractive index and light confinement which leads to low light loss and higher *Q*-factor [3,16,21]. For the tubular microcavity in our work, the most important loss terms are bulk adsorption (*Q*_mat_^-1^) and loss introduced by surfaced contaminants (*Q*_cont_^-1^): *Q*^-1^ = *Q*_mat_^-1^ + *Q*_cont_^-1^[5,18]. The adsorption of water molecules on the surface will increase the roughness of the tube wall as one kind of contaminant which magnifies *Q*_cont_^-1^ and consequently deteriorates the entire *Q*-factor. The desorption of water molecules, on the contrary, will enhance the *Q*-factor. Both the water molecule desorption and the increase of the tube wall thickness during ALD contribute to the enhancement of the *Q*-factor, as shown in Figure 2b.

## Conclusions

In summary, we have demonstrated that physisorption and chemisorption of water can influence the optical resonance in rolled-up Y_2_O_3_/ZrO_2_ tubular microcavity. Desorption of these two kinds of water molecules from the surface of the tube wall at high temperature can cause a blueshift of optical modes while additional coating of oxide layers with high refractive index leads to a redshift of the modes. Although both effects promote the *Q*-factor of the microcavity, the competition among them produces a bi-directional shift of the modes during the ALD process. Our current work demonstrates the feasibility of precisely modulating the modes of the rolled-up microcavity with a fine structure and high *Q*-factor. These discoveries may find potential applications in environmental monitoring. For instance, a humidity sensor using a tubular microcavity as a core component can be fabricated to detect the humidity variation of the environment.

## Abbreviations

AFM: Atomic force microscope; ALD: Atomic layer deposition; FWHM: Full width at half maximum; ML: Monolayer; PL: Photoluminescence.

## Competing interests

The authors declare that they have no competing interests.

## Authors’ contributions

GY and YM designed the study. JZ performed the experiments with help from JW. JZ, JW, GH, and YM contributed in drafting the manuscript. All the authors took part in the discussion of the results and edited and approved the manuscript.
